# Polysaccharides as Quality Marker to Rapid Profile for *Ophiocordyceps sinensis* by PXRD

**DOI:** 10.3390/molecules29133201

**Published:** 2024-07-05

**Authors:** Weien Wang

**Affiliations:** School of Chemistry and Chemical Engineering, Qinghai Normal University, Xining 810008, China; 2010044@qhnu.edu.cn; Tel.: +86-13997228516; Fax: +86-09715213524

**Keywords:** *Ophiocordyceps sinensis*, PXRD, intracellular polysaccharide, quality marker

## Abstract

Background: *Ophiocordyceps sinensis* has long been recognized as a mysterious and valuable traditional Chinese medicine but there has been little research on quality markers for *O. sinensis*. Purpose: This study looked into the potential of using powder X-ray diffractometry (PXRD) to analyze polysaccharides as a quality marker for *O. sinensis*. Study design: There were 16 different habitats of *O. sinensis* collected in Qinghai, Gansu, Sichuan, Yunnan, and Tibet. In addition, five different types of *Cordyceps* species were collected. The characteristic diffraction peaks of *O. sinensis* were determined and then matched with the characteristic diffraction peaks of intracellular polysaccharides obtained from *O. sinensis* to determine the attribution relationship of the characteristic diffraction peaks. Methods: *O. sinensis* powder’s X-ray diffraction pattern is determined by its composition, microcrystalline crystal structure, intramolecular bonding mechanism, and molecular configuration. After fractionation and alcohol precipitation of crude intracellular polysaccharide, mycelium crude intracellular polysaccharide (MCP) and fruiting body crude intracellular polysaccharide (FCP) were obtained and the fingerprint of *O. sinensis* was identified by the specific characteristic peaks of the X-ray diffraction pattern from intracellular polysaccharide. Results: The results indicated that the PXRD patterns of different populations of *O. sinensis* were overlaid well with 18 characteristic diffraction peaks obtained by microcrystalline diffraction. Moreover, the powder diffractograms as a fingerprint provided a practical identification of *O. sinensis* from other *Cordyceps* species. In addition, we detected that the powder diffractograms of intracellular polysaccharide MCP and MCP75 could be coupled with the PXRD of *O. sinensis*. Specifically, 18 characteristic diffraction peaks were identified as coming from MCP and MCP75 according to those interplanar crystal spacing, which matched well with those of PXRD of *O. sinensis*. Conclusions: PXRD spectra combined with an updated multivariable discriminant model were found to be an efficient and sensitive method for *O. sinensis* quality control. According to the findings of this study, PXRD should be further investigated for quality control assessments and plant extract selection trials.

## 1. Introduction

**Figure 1:**
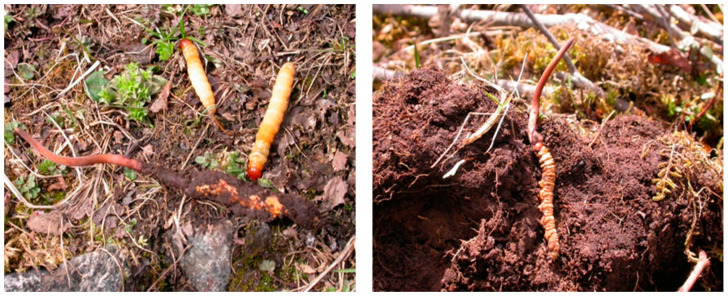
The photos of natural *O. sinensis* were taken by the author in Tongren County, Qinghai Province.

*Ophiocordyceps sinensis* (Berk.) Sacc. Sung et al. (Subphyla: Ascomycota; Class: Sordariomycetes; Order: Hypocreales; Family: Ophiocordycipitaceae; Genus: Ophiocordyceps Petch) is a kind of caterpillar fungus endemic to the vast region of the Qinghai-Tibetan plateau [1,2,3]. As a mysterious and precious traditional Chinese medicine, *O. sinensis* refers to a dried complex of the parasitic fungus growing on the larva of caterpillar (Family: Hepialidae). Basically, *O. sinensis* is a fungus–caterpillar complex consisting of the stromata of the fungus. The parasitic complex of the fungus and the caterpillar is found in the soil of alpine meadows, mountain shrub steppe, and shady slope of mountain steppe at an elevation of 3500∼5000 m. The *O. sinensis* complex is found in the Chinese provinces of Qinghai, Tibet, Sichuan, Yunnan, and Gansu, as well as three neighboring countries: Bhutan, India, and Nepal. According to a recent estimate, China accounts for more than 90% of its known production areas and more than 95% of its annual yield [4,5]. Moreover, official statistics have shown that the production of the *O. sinensis* complex in Qinghai was 200 tons in 2021. That accounts for more than 80% of its production in China. Figure 1 depicts photographs of natural O. sinensis taken by the author in Tongren County, Qinghai Province.

*O. sinensis* has been known and used in China for over 300 years for medication. The traditional medicine known as Chinese caterpillar fungus (commonly known as winter worm summer grass) has been widely used as a tonic food and traditional Chinese medicine to treat asthma and bronchial and lung inflammation, to strengthen the lungs and kidneys, and to increase energy while recovering from illness. According to Chinese tradition and the Chinese Pharmacopoeia, *O. sinensis* can “tonify the lung, replenish the kidneys, arrest bleeding, dissolve phlegm, for the treatment of chronic coughs, hyposexualities, spermatorrhea, low back and knee pain, spontaneous sweating, and restore strength after an illness” [6]. Current pharmacological studies show that *O. sinensis* has immunomodulatory, anticancer, antidiabetes, kidney protective, lung and liver protective, and antifatigue effects, improves stamina, and so on [7,8,9,10,11,12]. It is also an adaptogen in traditional Chinese medicine, which means it builds resistance to stress and boosts energy [13,14].

More than 400 species of Cordyceps have been reported in the world, including 120 species just in China [15]. As an indigenous fungus, the *O. sinensis* population has shown high genetic diversity in different habitat regions [16,17,18]. In view of this situation, in addition to classic characteristic identification and microscopic identification, high-pressure liquid phase fingerprints and near-infrared fingerprints are available; however, none of them contain quality markers [19,20,21,22]. We need to develop a simple and quick way of analyzing quality markers for *O. sinensis* quality control. In other words, it is necessary to identify the chemical markers of *O. sinensis* and then to develop a multi-index specific chemical profile that may distinguish *O. sinensis* from other Cordyceps. This will determine whether *O. sinensis* is present in Chinese patent medicine.

*Ophiocordyceps sinensis* has long been recognized as a mysterious and valuable traditional Chinese medicine but there has been little research on quality markers for *O. sinensis*. For *O. sinensis* quality control, we need to design a simple and efficient method of analyzing quality markers. To put it another way, the goal is to determine the chemical markers of *O. sinensis* and then create a multi-index unique chemical profile by PXRD that can distinguish *O. sinensis* from other *Cordyceps*. This will detect the presence of *O. sinensis* in Chinese patent medicine.

## 2. Results and Discussion

### 2.1. Study on the Fingerprint Characteristics of O. sinensis by Fourier Transform Infrared Spectroscopy

**Figure 2:**
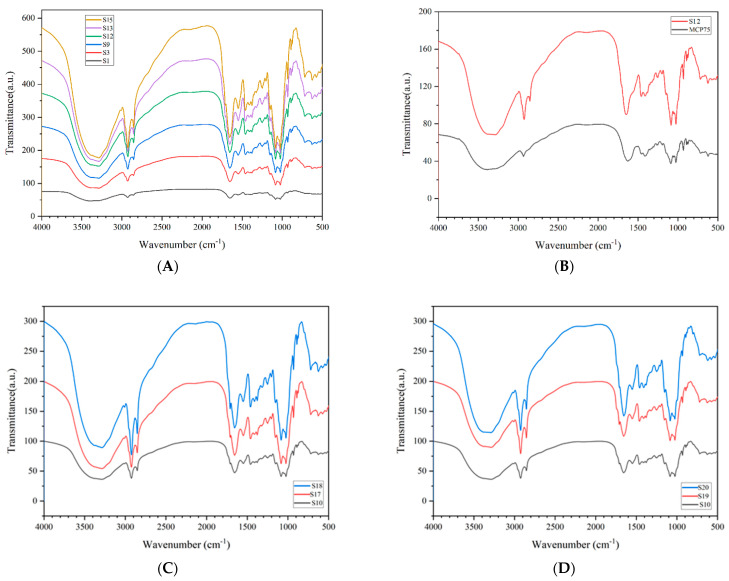
Comparative analysis of IR spectroscopy.

A Fourier transform infrared analysis of 21 Cordyceps samples was completed. There is some characteristic absorption in every analysis profile but without significant differences. The characteristic absorption peaks appear at 3288, 2925, 2854, 1655, 1548, 1458, 1410, 1249, 1151, 1084, 1024, 931, 890, 874, and 625 cm^−1^, and the comparative analysis of the IR spectroscopy is shown in Figure 2.

Part A shows the comparison of representative infrared spectroscopy between S1 Tongren, S3 Luqu, S9 Gande, S12 Zhiduo, S14 Heka, and S15 Diqing. Part B shows the comparison of representative infrared spectroscopy between S12 Zhiduo and MCP75. Part C shows the comparison of representative infrared spectroscopy between S10 Chenduo, S17 *Cordyceps gunnii,* and S18 *Cordyceps hawkesii.* Part D shows the comparison of representative infrared spectroscopy between S10 Chenduo, S19 *Cordyceps gracilis,* and S20 *Cordyceps militaris*.

**Figure 3:**
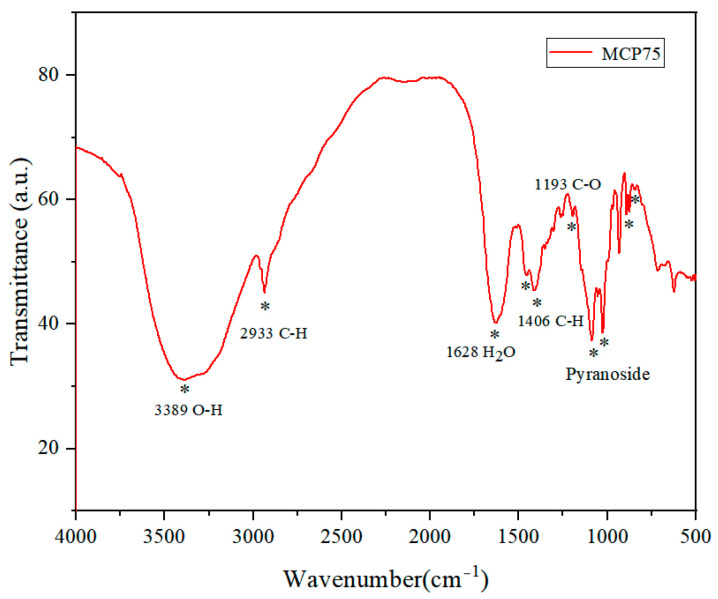
Infrared spectra of MCP75 from *O. sinensis* (* points to the characteristic absorption peak).

The infrared spectra of intracellular polysaccharides MCP75 and MCP85 are basically the same. Table 1 and Figure 3 show the infrared spectral characteristics of MCP75.

Figure 3 shows the infrared spectrum of MCP75. The intense peak located at approximately 3389 cm^−1^ was assigned to the O-H stretching vibrations of MCP75. The bands in the region of 2933 cm^−1^ were attributable to the C-H stretching vibration, while those in the region of 1628 cm^−1^ were due to associated water. The absorption bands at 1193 cm^−1^ were due to C-O stretching vibrations. The bands in the region of 1453 cm^−1^ and 1406 cm^−1^ were attributable to the C-H deformation vibration. Moreover, the characteristic absorption in the region of 1085 cm^−1^, 1053 cm^−1^, and 1025 cm^−1^ indicated pyranoside existing in polysaccharide MCP75. And the characteristic absorption at 890 cm^−1^ indicated β-configurations exist in polysaccharide MCP75 while the absorption bands at 844 cm^−1^ were ascribed to *α*-type glycosidic linkages [23,24].

Based on saccharide mapping, polysaccharides from various species of natural and cultured *Cordyceps* can be distinguished, implying that polysaccharides from different species of *Cordyceps* are made of different monosaccharides and structural characteristics. The partial acid hydrolysates of polysaccharides from natural *O. sinensis* were significantly similar in saccharide mapping, despite being clearly distinct from those of other *Cordyceps* species, including *C. gunnii*, *C. hawkesii*, cultured *C. militaris*, cultured *C. sinensis*, and *C. gracilis*. According to the current research, the major polysaccharides from natural *O. sinensis* were galactose, glucose, and mannose, with galactomannan as the backbone [25,26]. However, in our current research, it was difficult to distinguish different samples directly from IR spectrograms. It is worth noting that the infrared spectral profiles of polysaccharide MCP75 from native *O. sinensis* were nearly identical to those of all other native *O. sinensis* samples.

### 2.2. Study on Powder X-ray Diffraction (PXRD) Fingerprints of O. sinensis

**Figure 4:**
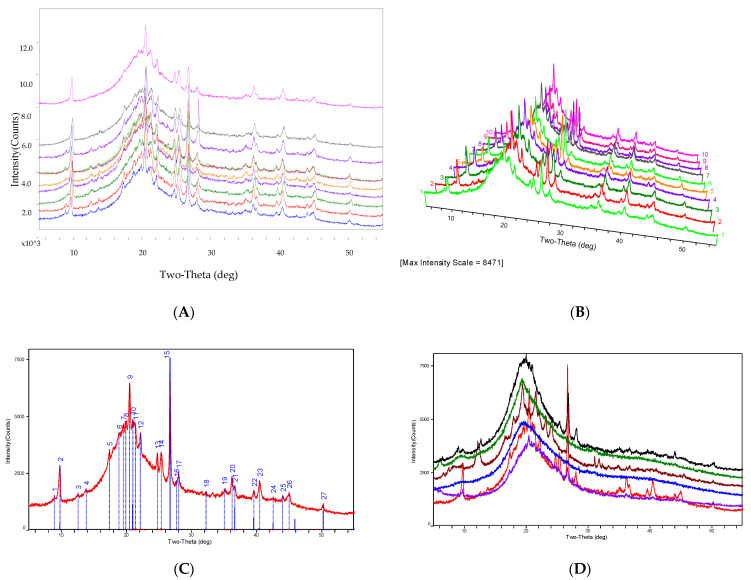
PXRD of *O. sinensis* and the different PXRD from other *Cordyceps* species.

The distinctive X-ray diffraction pattern of *O. sinensis* powder is determined by its composition, microcrystalline crystal form, intramolecular bonding mechanism, and molecular configuration. It has an exclusive corresponding relationship with all of the chemical components found in *O. sinensis* and the fingerprint of *O. sinensis* can be identified by the specific characteristic peaks of the X-ray diffraction pattern, and that was shown in Figure 4.

The experimental research discovered that the PXRD profile of *O. sinensis* from 16 different habitats can be essentially fitted, indicating that their chemical markers are consistent, as shown in Figure 4A–C. However, the PXRD profiles of S17, S18, S19, S20, and S21 exhibited amorphous diffraction characteristics, a lack of microcrystal diffraction characteristics spectra, and their PXRD patterns differed considerably from those of *O. sinensis* samples, as shown in Figure 4D.

Part A displayed a comparison of representative PXRD spectra from the following systems: S1 Tongren, S3 Luqu, S4 Songpan, S8 Maqin, S9 Gande, S11 Zaduo, S12 Zhiduo, S13 Naqu, S15 Diqing, and S16 Qilan (from bottom to top). Part B exhibited a comparison chat in 3D mode of exemplary PXRD spectra from S1 Tongren, S3 Luqu, S4 Songpan, S8 Maqin, S9 Gande, S11 Zaduo, S12 Zhiduo, S13 Naqu, S15 Diqing, and S16 Qilan (from bottom to top). Part C displayed sample S12′s PXRD spectrum along with its 27 distinctive diffraction peaks. Part D displayed a comparison of representative PXRD spectra from the following species: S12 Zhiduo (red line), S17 *C. gunnii* (purple line), S18 *C. hawkesii* (blue line), S19 *C. gracilis* (brown line), S20 *C. militaris* (green line), and S21 *Hirsutella sinensis* (black line) (from bottom to top).

#### 2.2.1. Determination of Related Chemical Markers in PXRD Spectrum of S12 Zhiduo

The authors present a crystallographic method for detecting bioactive compounds in raw polycrystalline materials and plant extracts. It is based on quick and simple overlays of simulated PXRD patterns from isolated chemical markers to empirically obtained diffractograms of the examined material with a content of at least 1%. Intracellular polysaccharide, mannitol, cordycepin, nucleoside, sitosterol, ergosterol, polypeptide, and other chemical components are found in *O. sinensis*, with intracellular polysaccharide and mannitol accounting for more than 1% of the total [23,27,28,29,30]. Mannitol is a common component among them but it is not what sets *O. sinensis* apart. Mannitol, for example, is present in lower plants as well as higher plants, including basidiomycetes, algae, and lichens. The best microcrystalline chemical components in *O. sinensis* that can induce a PXRD reaction are intracellular polysaccharide and mannitol. There is, however, no correlation between the PXRD spectrum of mannitol and that of *O. sinensis*. MCP75 and MCP contain chemical markers of *O. sinensis* according to the relationship between the PXRD spectrum of intracellular polysaccharide and the PXRD spectrum of *O. sinensis*.

##### The Homogeneity and Average Molecular Weight of the Intracellular Polysaccharide of *O. sinensis*

**Figure 5:**
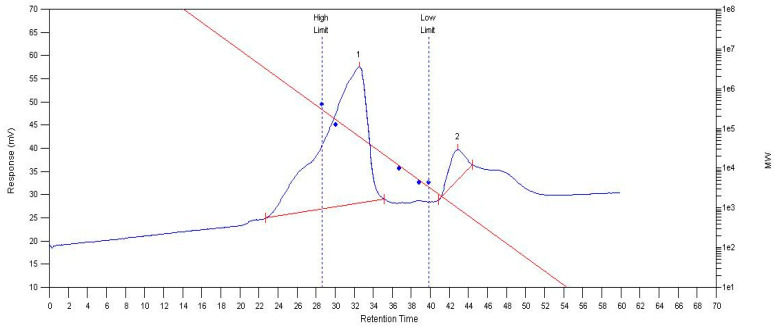
Molecular mass chromatogram of MCP75 from *O. sinensis* obtained by GPC. (The red line in the image represents the standard curve, whereas the blue line represents the molecular weight distribution. Peaks 1 and 2 indicate distinct polymer areas.)

MCP75 contains two parts of intracellular polysaccharide after separation and analysis with an Agilent PL-GPC 50; the average relative molecular weight (Mn) of part 1 is 167 kDa, the polydispersity index (PDI) is 2.31. The average relative molecular weight (Mn) of part 2 is 1031 Da and the polydispersity index (PDI) of part 2 is 1.07. A molecular mass chromatogram of MCP75 is shown in Figure 5.

MCP85 has a polydispersity index (PDI) of 2.62 and a number average relative molecular weight (Mn) of 106 kDa. The average relative molecular weight (Mn) of part 1 of FCP75 is 10.0 kDa, and the polydispersity index (PDI) is 18. FCP85 contains two portions of intracellular polysaccharide; part 1 has an average relative molecular weight (Mn) of 33.2 kDa and a polydispersity index (PDI) of 3.98. Part 2′s average relative molecular weight (Mn) is 1135 Da and its polydispersity index (PDI) is 1.14.

#### 2.2.2. MCP and MCP75 Fit for Comparable Chemical Markers in the S12 PXRD Spectrum

**Figure 6:**
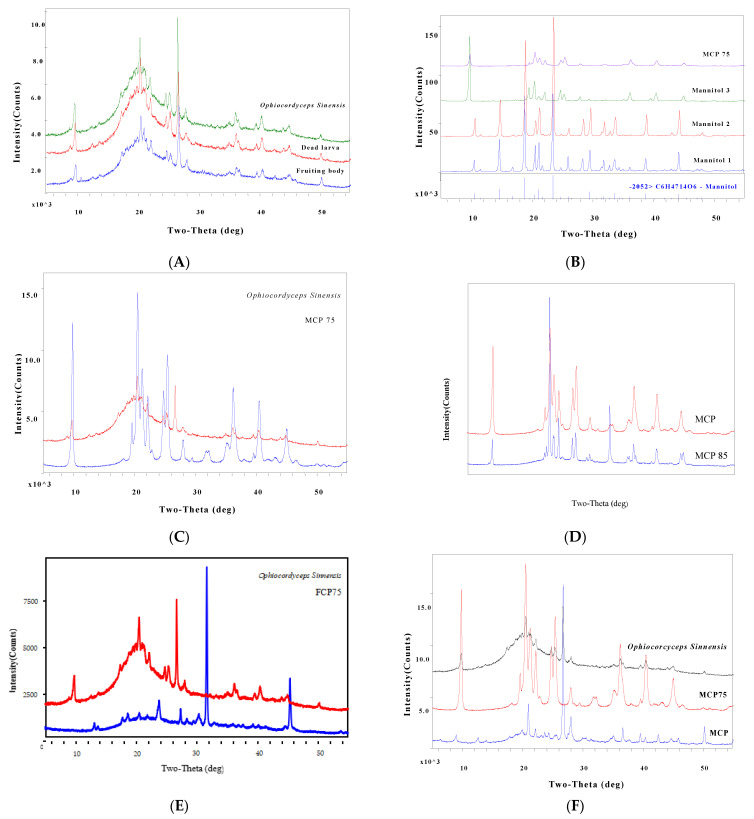
PXRD can be used to identify the quality marker for *O. sinensis*.

MCP75′s diffraction peak is shown to be closely related to that of *O. sinensis.* Combined with the diffraction connection of the MCP and *O. sinensis* peaks, we identified 18 unique diffraction peaks in the PXRD spectrum of *O. sinensis* as features associated with the intracellular polysaccharides MCP75 and MCP. The results of the analysis are depicted in Figure 6, Table 2, Table 3, Table 4 and Table 5.

Part A featured a comparison of representative PXRD spectra from fruiting bodies, dead larvae, and *O. sinensis*. Part B displayed a comparison of PXRD spectra of MCP75 and mannitol (D-Mannitol 3 isolated from *O. sinensis*), with the diffraction peak of mannitol standard not being well connected with that of MCP75. Part C demonstrated the comparison of PXRD spectra of MCP75 and *O. sinensis*, demonstrating that the diffraction peak of MCP75 is well correlated with that of *O. sinensis*. Part D displayed a comparison of PXRD spectra of MCP75 and MCP85, with the diffraction peak of MCP85 not being strongly connected with that of MCP75. Part E displayed a comparison chat of PXRD spectra of FCP75 and *O. sinensis*, with the diffraction peak of FCP75 not being strongly connected with that of *O. sinensis*. Part F demonstrated the comparison of PXRD spectra of MCP, MCP75, and *O. sinensis*, demonstrating that the diffraction peak of MCP and MCP75 can be highly ordered and associated with that of *O. sinensis*.

Specific features of intracellular polysaccharide MCP75 PXRD were expressed as *d*-(*I/I*_0_)%: 9.0176/100, 4.3329/99.1, 3.5037/69.0, 4.1797/53.3, 2.4793/45.4, 3.5929/43.1, 2.2276/40.6, 4.0116/37.0, 4.5347/26.7, 2.0112/23.7, 2.4520/19.5, 3.1907/14.7, 2.5545/13.3, 2.8134/7.7, 2.7743/7.5, 2.2752/3.9, 2.1025/3.1, 3.0276/3.0, 1.9513/2.7, and 1.8193/1.3.

Specific features of intracellular polysaccharide MCP PXRD were expressed as *d*-(*I/I*_0_)%: 3.3337/100, 4.2426/20.5, 3.1840/14.8, 1.8159/11.2, 2.4519/8.7, 2.2783/5.5, 2.1252/5.3, 4.0155/5.2, 9.9036/4.8, 2.5573/3.8, 3.7607/3.5, 3.6596/3.5, 7.0103/3.2, 4.4578/3.1, 3.2406/3.1, 1.9763/3.0, 6.3638/1.7, and 4.9682/1.6.

Specific features of *O. sinensis* from Zhiduo PXRD were expressed as *d*-(*I/I*_0_)%: 3.3385/100, 4.3327/38, 9.0188/28.6, 3.5119/17.5, 4.2306/15.3, 2.4780/15.1, 3.5930/14.6, 2.2276/14.6, 4.0148/14.5, 3.1817/12.1, 4.1533/10.4, 2.0138/7.9, 4.4582/6.5, 2.2752/6.0, 1.8145/6.0, 4.5438/5.4, 2.5600/5.0, 3.2222/3.9, 7.0315/3.5, 2.1236/3.3, 6.3952/3.2, and 2.7748/3.1. However, these 22 diffraction peaks are not shared by all populations of *O. sinensis* in this study, and the following are the frequent diffraction characteristics *d*-(*I/I*_0_)% (n = 16): 3.3325/100, 4.3344/66.2, 9.0296/53.6, 3.5114/31.3, 3.5956/26.7, 2.4790/25.8, 4.0091/25.2, 4.2396/24.8, 2.2284/24.1, 3.1810/21.7, 4.4582/15.7, 2.4507/13.2, 2.0148/11.8, 1.8158/9.9, 2.2768/9.6, 2.5578/9.1, 6.4306/7.7, and 2.1258/6.9. The results of the mean value of *d*-(*I/I*_0_)% (*n* = 16) are depicted in Table 5. These 18 diffraction peaks can be used as *O. sinensis* PXRD fingerprints from chemical markers MCP75 and MCP, which can be used for quality control of natural *O. sinensis*. Moreover, those powder diffractograms as fingerprints provided a practical identification of *O. sinensis* from other Cordyceps species. Other Cordyceps species’ diffraction characteristics are as follows:

The PXRD patterns of *C. militaris* and *Hirsutella sinensis* demonstrate an amorphous form, with diffraction patterns that differ markedly from normal *O. sinensis*. This demonstrates that cultured *Cordyceps* species might be attributed to the different polysaccharides strains or to being low isolated from natural *O. sinensis*. Specific features of *C. militaris* PXRD were expressed as *d*-(*I/I*_0_)%: 4.6054/100, 4.5349/92.2, 4.4445/62, 4.6724/47.7, and 3.3374/36.3. *Hirsutella sinensis* PXRD specific properties were expressed as *d*-(*I/I*_0_)%: 4.5674/100, 6.081/56.2, 9.3814/27.5, and 2.8913/27.4. PXRD analysis of *C. gunnii, C. hawkesii,* and *C. gracilis* revealed microcrystalline diffraction, although the typical diffraction lines and intensity differ from that of wild *O. sinensis*. There are a total of 26 diffraction peaks. PXRD characteristics of *C. gunnii* were expressed as *d*-(*I/I*_0_)%: 4.5670/100, 4.1147/99.6, 3.6840/76.2, 7.1787/71.5, 3.3360/60.7, 3.8431/55.3, 7.4048/34.3, 2.8766/34.3, 4.4587/33.1, 2.8120/29.7, 5.3488/24.6, 4.3442/22.0, 2.4468/17.2, 9.0517/15.6, 11.8723/14.5, 13.4103/13.3, 5.2692/12.9, 3.4092/12.8, 2.5629/12.6, 2.1985/12.6, 1.9919/11.8, 2.4820/11.3, 1.8150/11.3, 10.6189/11.1, 2.6882/10.9, and 3.2830/10.70. There are 20 diffraction peaks in all. *C. hawkesii* PXRD specific traits were expressed as *d*-(*I/I*_0_)%: 4.3455/100, 3.3406/97.1, 4.2501/58, 9.0902/55.3, 3.2456/46.4, 4.153/33.7, 4.0225/29.3, 2.4833/29.3, 3.6116/27.5, 2.8878/26.5, 2.4496.7/25.5, 2.0572/25, 3.0226/22.3, 9.2823/21.9, 2.2191/19.9, 2.230/19.6, 1.8183/17.4, 2.4432/17, 2.2794/16.4, and 2.0188/15.3. There are 19 diffraction peaks in all. *C. gracilis* PXRD characteristics were given as *d*-(*I/I*_0_)%: 3.3283/100, 4.4360/31.4, 3.1706/30.7, 4.5528/22.7, 6.9636/21.1, 9.7518/20.1, 2.0546/17.8, 3.5066/17.2, 4.2410/16.5, 4.6909/16.1, 4.3225/15, 2.4482/12.2, 2.5559/12.1, 1.8132/10.9, 6.446/10.2, 9.0347/8.6, 2.9914/8.5, 2.3744/8.4, and 2.2239/7.4.

## 3. Materials and Methods

### 3.1. Instrument and Software

In this study, the PXRD patterns were recorded at room temperature on a D/max 2500 pc (Rigaku, Yokyo, Japan) Diffractometer (CuKα beam, λ = 1.5405Å, beam generator at 40 Kv and 50 mA, RINT2000 wide angle goniometer, continuous scan mode, scan axis *θ*–2*θ*, scan speed 1.0°/min, data acquisition width and range of 0.02 and 5–55° 2θ, respectively). An infrared spectroscopy (FT-IR) measurement was performed on the Nexus infrared spectrophotometer (Thermoscientific, Waltham, MA, USA). Samples were dried at 55 °C in a vacuum oven for 48 h prior to tableting with KBr powder. Spectra were recorded in the range of 4000–400 cm^−1^. Gel permeation chromatography (Agilent PL-50, Santa Clara, CA, USA) was used to perform the measurement of molecular weight. A centrifuge (Hukang TGL20M, Shaoxing, China) was employed to separate proteins from polysaccharides. A rotary evaporator (EYELA OSB-2100, Yokyo, Japan) was employed to concentrate the extraction solution. Jade 9.8 (MDI Materials Data, Livermore, CA, USA) and Originpro 2021 (OriginLab, Northampton, MA, USA) were used to preprocess the spectral data.

### 3.2. Samples and Sample Pretreatment

**Figure 7:**
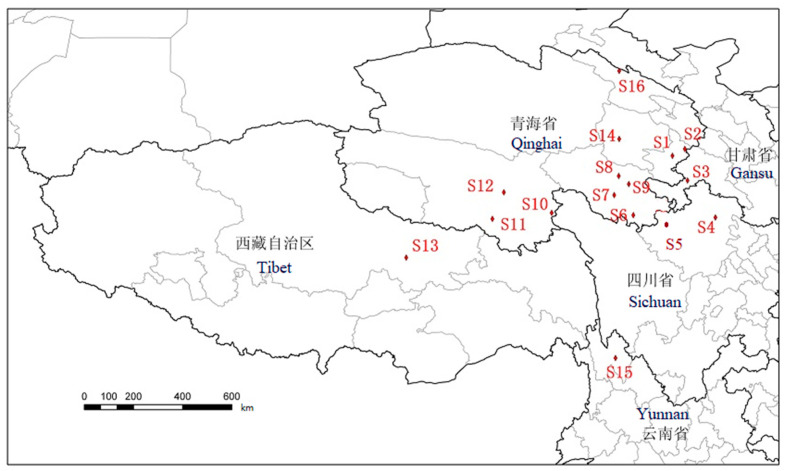
The sampling points of caterpillar fungus as S1 Tongren, S2 Hezuo, S3 Luqu, S4 Songpan, S5 Aba, S6 Banma, S7 Dari, S8 Maqin, S9 Gande, S10 Chenduo, S11 Zaduo, S12 Zhiduo, S13 Naqu, S14 Heka, S15 Diqing, and S16 Qilian.

There were 16 *O. sinensis* samples collected from 16 different habitats in Qinghai, Gansu, Sichuan, Yunnan, and Tibet. In addition, five different types of Cordyceps species were collected, including *Cordyceps gunnii* (Berk.) Berk., *Cordyceps hawkesii* (Gray) Cooke., *Cordyceps gracilis* (Grev.) Dur., *Cordyceps militaris* (L.) Link, and *Hirsutella sinensis*. The samples were pretreated as follows: Remove the mycoderm from fresh *O. sinensis*, brush away soil particles, dry them with silica gel powder, use 75% (*v*/*v*) ethanol for ultrasonic cleaning for 5 min, and dry them with absorbent paper. After drying for 6 h in an air drying oven at 60 °C, cut the dead larva from the fruiting body, seal and mark them, and then refrigerat them. The 16 samples taken from 16 different producing areas were numbered as follows: S1 Tongren, S2 Hezuo, S3 Luqu, S4 Songpan, S5 Aba, S6 Banma, S7 Dari, S8 Maqin, S9 Gande, S10 Chenduo, S11 Zaduo, S12 Zhiduo, S13 Naqu, S14 Heka, S15 Diqing, and S16 Qilian. And the five kinds of Cordyceps species samples were labeled as S17 *Cordyceps gunnii*, S18 *Cordyceps hawkesii*, S19 *Cordyceps gracilis*, S20 *Cordyceps militaris*, and S21 *Hirsutella sinensis* (cultured *Cordyceps sinensis*). All samples were kept in Qinghai Normal University and identified by Dr. Weien Wang. The sampling points of *O. sinensis* are shown in Figure 7. All voucher specimens were deposited at the Tibetan Medicine Research Laboratory of Qinghai Normal University.

Using a high-speed grinder, crush the dead larvae and fruiting bodies at 3 g each, then grind them with a high-throughput microgrinder. After sieving with 100, 250, and 325 mesh sieves, collect the PXRD analysis samples. The thickness of the PXRD analysis sample was 1 mm.

The D-Mannitol reference substances obtained from a reagent firm, such as D-Mannitol 1 and D-Mannitol 2. D-Mannitol 1 (Germany, Augsburg, Dr. Ehrenstorfer GmbH-Bgm, 99.0%), D-Mannitol 2, (UK, London, The British Drug House LTD.B.D.H. Laboratory Chemicals Group Poole, >99.0%), and D-Mannitol 3 were isolated from nature *O. sinensis*. All the reagents, solvents, and chemicals used in this work were analytical grade and used without further purification.

### 3.3. Preparation of Intracellular Polysaccharide from O. sinensis

A total of 400 g of dead larvae and 365 g of fruiting bodies were prepared to be crushed. They were extracted with CHCl_3_:CH_3_OH (1:1) 3 times at a liquid material ratio of 6:1 mL/g. After removing the fat-soluble components, the residue was extracted with a 30% ethanol solution for ultrasonic-assisted extraction. The extract was filtered by suction and then concentrated on the rotary evaporator at 56 °C and protein was removed by Sevag reagent (chloroform/1-butanol, *v*/*v* = 4:1) 15 times from the concentrated solution. The concentrated solution was added 4 times to the volume of absolute ethanol, which was stored in the refrigerator for 12 h. After centrifugation at 4000 r/min and freeze-drying, the mycelium crude intracellular polysaccharide (MCP) and fruiting body crude intracellular polysaccharide (FCP) yielded 3.1% and 3.4% of the total polysaccharide, respectively. After fractionation and alcohol precipitation of crude intracellular polysaccharide, 9.3 g MCP75 and 3.4 g MCP85 were recovered, respectively, along with 12 g FCP75 and 3.3 g FCP85.

### 3.4. Analysis of Intracellular Polysaccharides of O. sinensis by Gel Chromatography

The number- and weight-average molecular weights (M_n_ and M_w_) and molecular weight distributions (polydispersity index, PDI) M_w_/M_n_) were determined by GPC (gel permeation chromatography) measurements on Agilent Technologies PL-50 integrated GPC system, which was equipped with two Waters Hydrogel high-resolution columns (250 A) and columns (500 A). Water (flow rate of 0.5 mL/min) was used as eluent at 45 °C. Monodispersed dextran with a molecular weight range of 3300, 8100, 18,300, 100,000, and 273,000 was used to generate the calibration curve. The purified PS (polysaccharide) was dissolved in distilled water and prepared to create a solution of 0.5 mg/mL, filtered through 0.45 μm cellulose acetate filters (Angilent Captiva, Santa Clara, CA, USA), and then injected (50 μL) into the GPC system by maintaining the same flow rate and column temperature. Next, the retention times were plotted against their respective molecular weights. Thus, the weight-average molecular weight (Mw) and polydispersity index (Mw/Mn) were determined.

## 4. Conclusions

Natural *O. sinensis* resources have become more limited, falling far short of market demand. Counterfeit and inferior products are becoming increasingly common on the market. However, in the Chinese Pharmacopoeia, only the adenosine, which does not sufficiently reflect the clinical efficacy, is used as a quality control indicator. As a result, the development of an identification approach with high sensitivity and specificity is forthcoming. For this reason, we allow the phytochemical-specificity-effectiveness-Q-marker analytical strategy. Our findings showed that PXRD spectra paired with an adjusted multivariable discriminant model might be employed as an efficient and sensitive tool for *O. sinensis* quality control.

In summary, we find a quality marker to rapid profile for *O. sinensis* by PXRD, 18 diffraction peaks can be used as the PXRD fingerprint of *O. sinensis* from intracellular polysaccharide MCP75 and MCP, which can be used for quality control of nature *O. sinensis*. As a robust, direct, rapid, and nondestructive X-ray diffraction crystallography method to detect *O. sinensis*. It is valuable to apply it to the quality control of *O. sinensis*. Extensive work is still needed to know what is the structure of microcrystal MCP and MCP75 as well as whether their Miller indices match with those interplanar crystal spacing, and how we can find distinct differences in different genera of *O. sinensis* by PXRD. It is also of great significance to carry out a quantitative analysis of *O. sinensis* by PXRD.

## Figures and Tables

**Table 1:** Infrared spectra data of MCP75.

Ν (cm^−1^)	Absorption Peak
3389	O-H stretching vibrations
2933	C-H stretching vibration
1628	Bond water absorption peak
1193	C-O stretching vibrations
1406, 1453	C-H deformation vibration
1085, 1053, 1025	Pyranoside
890	β-type glycosidic linkages
844	*α*-type glycosidic linkages

**Table 2:** PXRD characteristic peaks of intracellular polysaccharides MCP75 from *O. sinensis*.

Peak No.	Peak Location (2*θ*)	D-Spacing (Å)	Height	Phase ID	Relative Intensity (*I*/*I*_0_%)
1	9.800	9.0176	11,703	galactomannan	100
2	19.560	4.5347	3119	galactomannan	26.7
3	20.480	4.3329	11,594	galactomannan	99.1
4	21.240	4.1797	6234	galactomannan	53.3
5	22.141	4.0116	4325	galactomannan	37
6	24.759	3.5929	5049	galactomannan	43.1
7	25.400	3.5037	8075	galactomannan	69.0
8	27.940	3.1907	1723	galactomannan	14.7
9	29.479	3.0276	350	-	3.0
10	31.780	2.8134	900	-	7.7
11	32.239	2.7743	878	galactomannan	7.5
12	35.101	2.5545	1555	galactomannan	13.3
13	36.200	2.4793	5308	galactomannan	45.4
14	36.619	2.4520	2284	galactomannan	19.5
15	39.578	2.2752	451	galactomannan	3.9
16	40.461	2.2276	4746	galactomannan	40.6
17	42.982	2.1025	362	-	3.1
18	45.040	2.0112	2778	galactomannan	23.7
19	46.501	1.9513	320	-	2.7
20	50.097	1.8193	154	-	1.3

Note: - indicates that there is no corresponding phase identification.

**Table 3:** PXRD characteristic peaks of intracellular polysaccharides MCP from *O. sinensis*.

Peak No.	Peak Location (2*θ*)	D-Spacing (Å)	Height	Relative Intensity (*I*/*I*_0_%)
1	8.922	9.9036	721	4.8
2	12.617	7.0103	479	3.2
3	13.904	6.3638	262	1.7
4	17.839	4.9682	240	1.6
5	19.337	4.5864	199	1.3
6	19.901	4.4578	465	3.1
7	20.921	4.2426	3076	20.5
8	22.119	4.0155	780	5.2
9	23.638	3.7607	528	3.5
10	24.301	3.6596	523	3.5
11	26.719	3.3337	15,006	100
12	27.502	3.2406	461	3.1
13	28.000	3.1840	2226	14.8
14	35.060	2.5573	573	3.8
15	36.619	2.4519	1311	8.7
16	39.521	2.2783	818	5.5
17	42.501	2.1252	800	5.3
18	45.879	1.9763	451	3.0
19	50.198	1.8159	1675	11.2

Note: - indicates that there is no corresponding phase identification.

**Table 4:** Determination results of PXRD fingerprint common peaks in *O. sinensis* from S12 Zhiduo.

Peak No.	Peak Location (2*θ*)	D-Spacing (Å)	Height	Phase ID	Relative Intensity (*I*/*I*_0_%)
1	8.922	9.9030	183	-	3.3
2	9.799	9.0188	1564	MCP75	28.6
3	12.578	7.0315	192	MCP	3.5
4	13.836	6.3952	176	MCP	3.2
5	17.381	5.0979	464	-	8.5
6	18.855	4.7027	202	-	3.7
7	19.520	4.5438	297	MCP75	5.4
8	19.899	4.4582	354	-	6.5
9	20.481	4.3327	2082	MCP75	38.0
10	20.981	4.2306	837	MCP	15.3
11	21.376	4.1533	569	MCP75	10.4
12	22.123	4.0148	795	MCP75	14.5
13	24.759	3.5930	798	MCP 75	14.6
14	25.340	3.5119	957	MCP 75	17.5
15	26.680	3.3385	5473	MCP	100.0
16	27.661	3.2222	216	MCP	3.9
17	28.020	3.1817	663	MCP 75	12.1
18	32.233	2.7748	168	MCP75	3.1
19	35.023	2.5600	275	MCP75	5.0
20	36.222	2.4780	828	MCP75	15.1
21	36.640	2.4506	456	MCP75	8.3
22	39.578	2.2752	330	MCP75	6.0
23	40.460	2.2276	797	MCP75	14.6
24	42.534	2.1236	133	MCP	3.3
25	43.982	2.0570	214	-	3.9
26	44.978	2.0138	430	MCP75	7.9
27	50.239	1.8145	327	MCP	6.0

Note: - indicates that there is no corresponding phase identification.

**Table 5:** The frequent diffraction characteristics peaks in *O. sinensis* from 16 different habitats.

Code	1 *d*-(*I/I*_0_)%	2 *d*-(*I/I*_0_)%	3 *d*-(*I/I*_0_)%	4 *d*-(*I/I*_0_)%	5 *d*-(*I/I*_0_)%	6 *d*-(*I/I*_0_)%	7 *d*-(*I/I*_0_)%	8 *d*-(*I/I*_0_)%	9 *d*-(*I/I*_0_)%
**S1 Tongren**	9.0174/64.1	6.4199/6.5	4.4417/16.9	4.3287/64.9	4.2354/11.5	4.0111/25.1	3.5901/27.7	3.5038/34.4	3.3286/100
**S2 Hezuo**	8.9636/36.2	6.4045/4.8	4.4404/10.5	4.3125/45.3	4.2151/26.8	4.0011/15.8	3.5869/16.8	3.4957/23.7	3.3262/100
**S3 Luqu**	9.0568/72.6	6.4655/8.6	4.4795/15.7	4.3373/84.7	4.2350/27.7	4.0117/32.6	3.5985/31.6	3.5146/34.8	3.3337/100
**S4 Songpan**	9.0730/13.0	6.4683/2.4	4.4711/5.3	4.3416/16.7	4.2429/21.3	4.0157/8.6	3.6016/6.0	3.5227/10.5	3.3361/100
**S5 Aba**	9.0550/19.7	6.4581/5.6	4.4755/8.2	4.3332/29.2	4.2387/24.1	4.0110/15.8	3.6036/9.0	3.5173/16.1	3.3359/100
**S6 Banma**	9.0378/45.7	6.4119/6.4	4.4584/15.7	4.3332/51.4	4.2345/17.9	4.0117/20.3	3.5982/20.1	3.5146/25.6	3.3337/100
**S7 Dari**	9.0571/77.4	6.4501/10.6	4.4630/13.6	4.3411/86.7	4.2590/25.0	4.0153/32.5	3.6039/34.6	3.5198/43.2	3.3383/100
**S8 Maqin**	8.8564/38.7	6.3760/3.6	4.4271/7.9	4.2916/49.0	4.1952/20.6	3.9727/21.4	3.5728/18.7	3.4770/23.3	3.3069/100
**S9 Gande**	9.1292/67.5	6.4847/17.7	4.4804/25.4	4.3578/89.3	4.2462/30.5	4.0227/36.3	3.6072/34.1	3.5310/43.9	3.3481//100
**S10 Chenduo**	9.1287/78.1	6.4603/10.1	4.4642/19.3	4.3501/87.2	4.2550/16.0	4.0256/29.5	3.6099/35.0	3.5308/35.8	3.3435/100
**S11 Zaduo**	8.9098/33.6	6.3476/4.6	4.4315/10.3	4.3000/50.6	4.2147/25.7	3.9800/18.5	3.5784/17.6	3.4876/24.7	3.3165/100
**S12 Zhiduo**	9.0188/28.6	6.3952/3.2	4.4582/6.5	4.3327/38.0	4.2306/15.3	4.0148/14.5	3.5930/14.6	3.5119/17.5	3.3385/100
**S13 Naqu**	9.0752/77.7	6.4683/11.8	4.4702/19.7	4.3454/100	4.1762/33.9	4.0186/38.7	3.6044/44.6	3.5201/44.8	3.3360/93.0
**S14 Heka**	9.0001/64.2	6.4293/11.2	4.4406/26.2	4.3244/78.1	4.2221/31.5	4.0041/26.3	3.5845/35.3	3.5091/37.0	3.3261/100
**S15 Diqing**	9.0750/81.5	6.4375/5.9	4.4715/22.5	4.3417/97.7	4.1838/32.2	4.0187/34.7	3.6070/37.2	3.5174/44.0	3.3384/100
**S16 Qilian**	9.0189/58.8	6.4131/10.7	4.4574/27.2	4.3368/73.0	4.2335/24.6	4.0113/33.1	3.5959/24.3	3.5093/45.0	3.3335/100
$\bar{x}$	9.0296/53.6	6.4306/7.7	4.4582/15.7	4.3344/66.2	4.2396/24.8	4.0091/25.2	3.5956/26.7	3.5114/31.3	3.3325/100
**Code**	**10** ***d*-(*I/I*_0_)%**	**11** ***d*-(*I/I*_0_)%**	**12** ***d*-(*I/I*_0_)%**	**13** ***d*-(*I/I*_0_)%**	**14** ***d*-(*I/I*_0_)%**	**15** ***d*-(*I/I*_0_)%**	**16** ***d*-(*I/I*_0_)%**	**17** ***d*-(*I/I*_0_)%**	**18** ***d*-(*I/I*_0_)%**
**S1 Tongren**	3.1773/24.1	2.5547/7.5	2.4767/25.7	2.448/13.5	2.2750/10.8	2.2266/24.1	2.1207/6.1	2.0136/15.2	1.8158/11.0
**S2 Hezuo**	3.1685/26.1	2.5490/8.2	2.4755/16.2	2.4455/11.2	2.2727/9.6	2.2255/18	2.1187/5.8	2.0087/8.5	1.8125/11.7
**S3 Luqu**	3.1861/27.9	2.5616/8.8	2.4794/31.6	2.4558/15.3	2.2773/10.0	2.2318/27.2	2.1244/7.7	2.0153/15.7	1.8158/7.7
**S4 Songpan**	3.1905/15.4	2.5632/4.8	2.4820/6.3	2.4533/8.1	2.2795/5.8	2.2349/7.4	2.1254/5.6	2.0155/4.1	1.8165/11.8
**S5 Aba**	3.1819/16.1	2.5572/6.2	2.4781/11.7	2.4518/10.6	2.2773/6.1	2.2297/12.1	2.1252/6.3	2.0281/3.2	1.8152/11.8
**S6 Banma**	3.1840/22.6	2.5560/7.6	2.4794/20.2	2.4531/12.0	2.2773/8.8	2.2297/20.2	2.1261/6.7	2.0113/9.5	1.8152/9.8
**S7 Dari**	3.2020/22.3	2.5670/8.8	2.4820/30.2	2.4531/12.6	2.2806/9.9	2.2276/29.9	2.1253/5.9	2.0179/18.7	1.8159/11.2
**S8 Maqin**	3.1619/15.5	2.5490/7.7	2.4687/20.4	2.4404/10.1	2.2662/4.9	2.2182/19.5	2.1164/5.1	2.0265/5.8	1.8172/5.6
**S9 Gande**	3.1931/20.5	2.5662/11.2	2.4847/34.2	2.4544/14.7	2.2817/10.8	2.2318/31.1	2.1253/6.5	2.0197/14.0	1.8197/11.2
**S10 Chenduo**	3.1932/20.8	2.5598/12.1	2.4873/33.5	2.4559/19.3	2.2785/9.5	2.2349/30.5	2.1329/6.5	2.0179/16.6	1.8186/11.4
**S11 Zaduo**	3.1615/23.4	2.5502/8.7	2.4726/20.3	2.4428/12.1	2.2695/9.5	2.2213/18.0	2.1152/5.8	2.0095/11.0	1.8104/11.7
**S12 Zhiduo**	3.1817/12.1	2.5600/5.0	2.4780/15.1	2.4506/8.3	2.2752/6.0	2.2276/14.6	2.1063/5.0	2.0138/7.9	1.8145/6.0
**S13 Naqu**	3.1907/19.9	2.5596/10.0	2.4820/40.0	2.4530/13.0	2.2818/12.6	2.2308/35.0	2.1593/13.5	2.0179/21.1	1.8178/7.6
**S14 Heka**	3.1793/24.8	2.5515/9.4	2.4741/33.8	2.4457/16.0	2.2785/10.8	2.2255/29.8	2.1221/9.2	2.0137/14.5	1.8132/12.7
**S15 Diqing**	3.1886/31.2	2.5643/11.5	2.4847/34.6	2.4544/16.8	2.2827/9.6	2.2318/35.8	2.1259/5.2	2.0162/16.3	1.8165/7.2
**S16 Qilian**	3.1775/27.9	2.5602/14.4	2.4780/29.7	2.4533/16.9	2.2751/18.2	2.2276/33.0	2.1236/7.5	2.0122/18.4	1.8192/10.2
$\bar{x}$	3.1810/21.7	2.5578/9.1	2.4790/25.8	2.4790/25.8	2.2768/9.6	2.2284/24.1	2.1258/6.9	2.0148/11.8	1.8158/9.9

## Data Availability

All the data are contained within the article.

## Abbreviations

Fruiting body crude intracellular polysaccharide (FCP); mycelium crude intracellular polysaccharide (MCP); Ophiocordyceps sinensis (O. sinensis); powder X-ray diffractometry (PXRD); polydispersity index (PDI); gel permeation chromatography (GPC).
